# Glycogenome expression dynamics during mouse C2C12 myoblast differentiation suggests a sequential reorganization of membrane glycoconjugates

**DOI:** 10.1186/1471-2164-10-483

**Published:** 2009-10-20

**Authors:** Mathilde Janot, Aymeric Audfray, Céline Loriol, Agnès Germot, Abderrahman Maftah, Fabrice Dupuy

**Affiliations:** 1 INRA, UMR 1061 Unité de Génétique Moléculaire Animale, Université de Limoges, Faculté des Sciences et Techniques, 123 Avenue A. Thomas, 87060 Limoges, France

## Abstract

**Background:**

Several global transcriptomic and proteomic approaches have been applied in order to obtain new molecular insights on skeletal myogenesis, but none has generated any specific data on glycogenome expression, and thus on the role of glycan structures in this process, despite the involvement of glycoconjugates in various biological events including differentiation and development. In the present study, a quantitative real-time RT-PCR technology was used to profile the dynamic expression of 375 glycogenes during the differentiation of C2C12 myoblasts into myotubes.

**Results:**

Of the 276 genes expressed, 95 exhibited altered mRNA expression when C2C12 cells differentiated and 37 displayed more than 4-fold up- or down-regulations. Principal Component Analysis and Hierarchical Component Analysis of the expression dynamics identified three groups of coordinately and sequentially regulated genes. The first group included 12 down-regulated genes, the second group four genes with an expression peak at 24 h of differentiation, and the last 21 up-regulated genes. These genes mainly encode cell adhesion molecules and key enzymes involved in the biosynthesis of glycosaminoglycans and glycolipids (neolactoseries, lactoseries and ganglioseries), providing a clearer indication of how the plasma membrane and extracellular matrix may be modified prior to cell fusion. In particular, an increase in the quantity of ganglioside G_M3 _at the cell surface of myoblasts is suggestive of its potential role during the initial steps of myogenic differentiation.

**Conclusion:**

For the first time, these results provide a broad description of the expression dynamics of glycogenes during C2C12 differentiation. Among the 37 highly deregulated glycogenes, 29 had never been associated with myogenesis. Their biological functions suggest new roles for glycans in skeletal myogenesis.

## Background

Myogenesis is a complex process which leads muscle progenitor cells to proliferate and then differentiate into myotubes. This process is strongly controlled by the spatio-temporal expression of myogenic regulatory factors (MRFs) - MyoD, Myf5, myogenin and Mrf4 (or Myf6) [1,2] - and by several transcription factors of the myocyte enhancer factor-2 (MEF2) family [3]. Their expression defines different stages in the myogenic process: myoblast proliferation, cell-cycle withdrawal, cell fusion to form myotubes, and the maturation of myotubes into myofibers. MRFs are members of the bHLH (basic Helix-Loop-Helix) protein family [4]. They cooperate with MEF2 transcription factors to mediate the transcription of muscle-specific genes [5]. bHLH proteins also form heterodimers with E proteins [6,7], enabling binding to the E-box consensus DNA sequence [8] and the transcription of specific skeletal muscle genes, such as the myosin heavy chain gene [9].

As well as myogenic factors, myogenesis involves other molecular actors such as embryonic fibroblast growth factor (eFGF), cadherins, members of the cadherin-associated immunoglobulin superfamily such as CDO (CAM (Cell Adhesion Molecule)-related/down-regulated by oncogenes), BOC (brother of CDO) [10], neogenin [11] and p38 MAP kinase [12]. These are the classic molecules involved in cell interactions and signaling. In order to monitor the expression of these actors, several studies have exploited the development of high-throughput gene expression profiling using microarrays and proteomic approaches. Recent microarray studies on C2C12 cells, mouse myoblasts that can differentiate into myotubes, have afforded a broad molecular description of myogenesis and identified sets of genes that display transcriptional variations in expression between proliferating and differentiating cells [13-16]. These studies identified some genes, as *Zfp-51 *and *Ptger4*, which were not previously associated with skeletal myogenic differentiation. Some proteomics studies on developing myotubes have partially confirmed and completed these microarray-based studies by providing evidence for the involvement of transcription regulators, signaling factors, phospho-proteins and adhesion molecules, as well as novel non-characterized proteins (Riken clones and unnamed proteins) in skeletal muscle development and contractility [17,18].

The plasma membrane and extracellular matrix (ECM) of myoblasts, like those of other eukaryotic cells, are rich in glycoproteins and glycolipids. Despite all the data generated by transcriptomic and proteomic studies, little information is available on the role of glycoconjugates in myogenesis. The principal reason for this lies in the weak expression of glycogenes which is hardly detectable using pan-genomic microarrays. Nevertheless, some proteoglycans of the ECM, *e.g*. syndecans, have been shown to play different roles in myogenesis [19,20]. Inhibition of their synthesis halts myoblast proliferation and fusion independently of the expression of the myogenic bHLH factor. In the same way, blocking *N*-glycan synthesis impairs myoblast fusion [21] and the *in vivo *invalidation of *Mgat1*, a gene involved in the synthesis of complex *N*-glycans, generates mouse embryo death *in utero *[22]. Conversely, NCAM1 *O*-glycosylation promotes myoblast fusion [23,24]. Glycolipids also play key roles in cell differentiation [25,26]. They appear to be involved in muscle development, since their membrane levels are altered during G7 and G8 myoblast fusion, with an increase in gangliosides and neutral glycolipid synthesis [27]. In other myogenic cell lines, changes have been observed in the activities of the glycosyltransferases that contribute to glycolipid synthesis [28].

In order to clarify the potential roles of glycosylation in myogenesis, quantitative real-time RT-PCR was used to analyze the expression of 375 glycogenes (that account for more than 60% of the glycogenome) in differentiating mouse C2C12 cells. Seventy-four percent of the genes (276 genes) were expressed during C2C12 cell differentiation: 181 of them were invariant while 37 displayed up- or down-regulations of more than 4-fold. These genes were clustered in three main groups. The first cluster contained genes with gradually decreasing quantities of transcripts. In the second set of genes, transcript levels reached a maximum at 24-48 h of differentiation and then decreased, while those in the third cluster increased throughout differentiation. The functions controlled by the clustered genes, as a function of their group, highlighted how the myoblast cell membrane and ECM could be modified for cell fusion during C2C12 differentiation. For the first time, this study provides a general framework for glycogene expression during the onset of *in vitro *myogenesis.

## Results and Discussion

The combined use of cell lines and microarrays offers a major opportunity to study gene expression patterns and/or dynamics during different physiological and pathological processes. However, the substantial findings generated by the use of pangenomic microarrays have generally been difficult to interpret in terms of the gene regulation controlling biological functions. In this study, we chose to explore the expression dynamics of just one part of the mouse genome, called the 'glycogenome', in the context of myogenesis. For this purpose, we first of all standardized the experimental conditions for the differentiation of C2C12 (a mouse myogenic cell line), and analyzed the expression of myogenic markers by quantitative real-time RT-PCR. The expression of 375 glycogenes was then monitored in differentiating C2C12 cells using quantitative real-time RT-PCR with TLDA (TaqMan Low Density Array, see Methods section). Highly deregulated genes were next clustered as a function of their expression profiles. Their functions were analyzed and used to suggest new roles for glycoconjugates in myogenic differentiation.

### The expression of MRF and marker genes is consistent with C2C12 cell differentiation

**Figure 1:**
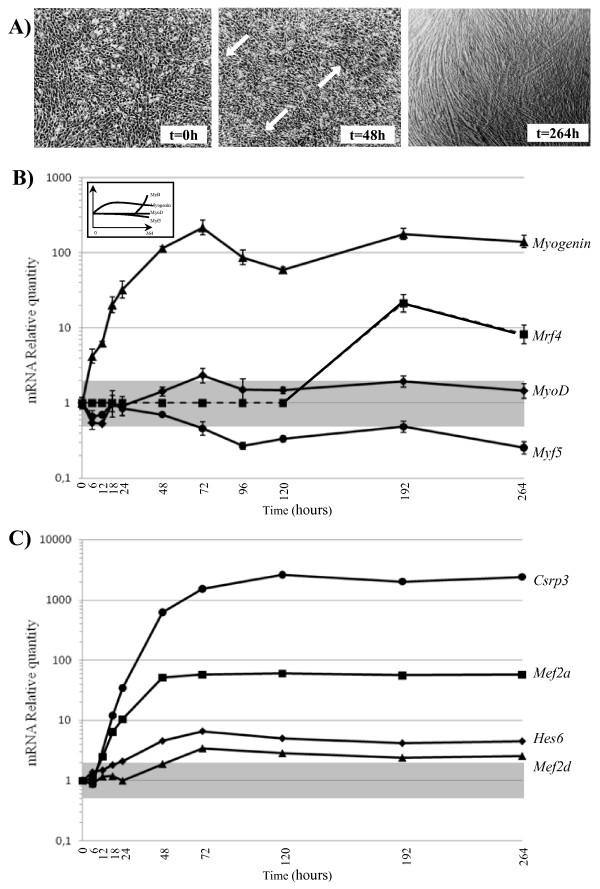
**Time course of C2C12 differentiation**. (A) C2C12 cells were placed in differentiation medium when culture reached 80% of confluence (t = 0 h). After 48 hours, the first myotubes, indicated by arrows, had clearly formed and after 11 days (t = 264 h), most of the cells had merged into myotubes. C2C12 RNA was extracted at several differentiation time points and tested for the presence of (B) mRNA from the myogenic markers *Myf5*, *MyoD*, *Myogenin *and *Mrf4 *and (C) mRNA from the muscle-specific markers *Csrp3*, *Hes6*, *Mef2a *and *Mef2d*. Transcription levels are expressed as relative quantities (RQ) compared to the initiation of differentiation (t = 0 h). The grey area includes no significant variations (RQ < ± 2). For (B), standard deviations were calculated on three separate experimental values. No significant transcriptional expression of *Mrf4 *was detected before t = 192 h (Ct>33, dotted line). The top inset presents the standard expression patterns of the four myogenic markers *Myf5*, *MyoD*, *Myogenin *and *Mrf4 *as found in the literature.

When cultured *in vitro*, C2C12 myoblasts start to differentiate following serum deprivation. The first myotubes appeared 48 hours after serum starvation and a maximum of mature multinucleated cells was obtained after 11 days in the differentiation medium (Figure 1A). Expression levels of the four MRFs (*Mrf4, Myf5, MyoD *and *myogenin*) and four marker genes (*Csrp3*, *Hes6*, *Mef2a *and *Mef2d*) known for their involvement in myogenic differentiation [29-31], were determined by quantitative real-time RT-PCR at different time points following the induction of C2C12 differentiation.

*MyoD, Myf5 *and *myogenin *genes were expressed throughout C2C12 differentiation (Figure 1B). *MyoD *mRNA levels only changed slightly, regardless of the time elapsing after the start of differentiation. Beyond t = 48 h, the expression of *Myf5 *decreased more than two-fold and remained down-regulated, while the *myogenin *gene was up-regulated (~100-fold). For *Mrf4*, transcripts were only detected at t = 192 h. Therefore, the expression profiles of myogenic regulatory factors during C2C12 differentiation were in agreement with their expression patterns (top diagram inset in Figure 1B) described in the literature [15,16,32,33].

Expression of the muscle transcription factors *Mef2a *and *Mef2d *increased as from 6 h of differentiation to reach 60-fold for *Mef2a *and 3.4-fold for *Mef2d *at the end of the experiment (Figure 1C). Their expression was in line with their myogenic activator roles [29]. Interestingly, the increase in *Hes6 *expression started at t = 6 h of differentiation and reached 6.5-fold after 72 h. As demonstrated elsewhere [31], this last result argued in favor of *Hes6 *involvement at the onset of C2C12 differentiation and more generally of the myogenic process. Unlike the *Hes *and *Mef *genes, *Csrp3 *expression was first detected at t = 18 h of differentiation and increased to reach a peak at t = 120 h (Figure 1C). The expression profile of *Csrp3*, encoding the LIM protein, correlated with its activator function of C2C12 differentiation. Indeed, it has been showed that LIM protein is not necessary for myoblast proliferation but plays a key role in upcoming myogenic differentiation [30]. Thus, the transcriptional expression profiles of both myogenic marker genes and MRFs genes attested to the accurate time course of C2C12 differentiation.

### Most glycogenes are expressed during the onset of C2C12 differentiation

The glycogenome refers to all genes involved in glycosylation. It includes ~600 genes and accounts for ~2 percent of the mouse genome. The expression of 375 glycogenes was analyzed during the first 72 h of C2C12 differentiation, when the first myotubes are formed. These 375 glycogenes account for more than 60% of the mouse genes known to be related to glycosylation (Table 1). The proteins encoded by these genes belong to glycosyltranferases, glycosidases, lectins, sulfotranferases or proteins involved in sugar metabolism or transport [see Additional file 1]. Given the known weak expression of most glycogenes, their expression patterns were determined by quantitative real-time RT-PCR using the TLDA technology which allows the simultaneous analysis of 375 selected genes [see Additional file 2].

**Table 1:** Data summary of *Mus musculus *glycogene expression during C2C12 differentiation.

**Protein function**	**Number of known genes^1^**	**Number of analyzed genes^2^**	**Number of expressed genes^3^**	**Genes with 2× up- or down-regulation^4^**	**Genes with 4× up- or down-regulation^4^**
Glycosyltransferase	209	146 (70%)	108 (74%)	37 (34%)	12 (11%)
Glycosidase	75	53 (71%)	47 (89%)	8 (17%)	2 (4%)
Sugar carrier	34	26 (76%)	19 (73%)	7 (37%)	3 (16%)
Translocase	2	2 (100%)	2 (100%)	0 (0%)	0 (0%)
Sugar metabolism	28	24 (86%)	23 (96%)	4 (17%)	0 (0%)
Lectin	193	102 (53%)	58 (57%)	29 (50%)	15 (26%)
Sulfotransferase	53	22 (42%)	19 (86%)	10 (53%)	5 (26%)

**Total**	**594**	**375 **(63%)	**276 **(74%)	**95 **(34%)	**37 **(10%)

^1^The number of known genes was determined in the MGI database [61] according to their functions.

^2^The number of analyzed genes, except for the sulfotransferase family, corresponded to assays available from the manufacturer. For sulfotransferase genes, 22 probes were chosen from the 35 available. Values in brackets indicate percentages of analyzed genes compared to known genes.

^3^Number of expressed genes corresponded to significantly expressed genes for at least one kinetic point according to their mRNA quantification by qRT-PCR (Ct<33). Values in brackets indicate percentages of expressed genes compared to analyzed genes.

^4^Up- and down-regulations correspond to variations in transcript quantity with a minimum factor of 2 or 4. Values in brackets correspond to percentages of deregulated genes compared to expressed genes.

Three-quarters of the genes analyzed were expressed (Table 1): 276 genes displayed significant quantities of transcripts (Ct ≤ 33) during at least one point of the differentiation time course. Among the 375 glycogenes of this study, 202 genes were also analyzed in Tomczak *et al*. study [16]. The microarray and TLDA approaches gave similar results for 91 genes, 43 were expressed and 48 unexpressed. For the remaining common genes (111), only TLDA revealed significant expression levels. This could be explained by the methodologies employed, insofar as microarray techniques are less precise and sensitive than quantitative real-time RT-PCR [34].

Among the genes expressed, 34% had a minimum 2-fold modification of their expression for at least one kinetic time, and 10% displayed a variation of at least 4-fold (Table 1). The significant number of glycogenes thus regulated underlined the critical function of glycosylation in this differentiation process. Lectin genes appeared to be regulated preferentially, because only 57% of them were expressed, compared to 73% or more for the other gene families. Within each glycogene family, it is interesting to note that no correlation was observed between the number of genes analyzed and the number of those regulated. Indeed, glycosyltransferase genes accounted for about 40% of analyzed genes and only 11% of them displayed an mRNA variation of more than 4-fold. At the same time, ~50% of lectin and sulfotransferase genes, representing ~27% and ~6% of analyzed genes respectively, were significantly modified in terms of their expression. In addition, no glycogene sub-family, such as fucosyltransferases or sialyltransferases, was preferentially repressed or expressed.

Genes displaying more than 4-fold variation (37 genes) were distributed into four groups according to their glyco-family (Table 1). The first group included lectin and sulfotransferase genes (26% of them with significant mRNA variations), the second contained glycosyltransferase and sugar carrier genes (11-16% deregulated), the third included glycosidase genes (only 4% of genes deregulated), and the final group comprised translocase and sugar metabolism genes in which no gene displayed a variation in mRNA expression. Thus, a large proportion of the modifications to glycogene expression that occurred during C2C12 differentiation mainly seemed to affect proteins giving rise to the glycans or lectins required for cell contacts. These results are consistent with the cellular events involved in myotube formation, *i.e*. cell interactions and fusions.

Among the genes analyzed, 99 were poorly or not expressed. Their corresponding mRNA were not detected (Ct = 40) or not significantly quantified (Ct>33). These genes encoded proteins involved in physiological processes unrelated to myogenesis. For example, *Has3 *encodes a hyaluronan synthase which is active in hyaluronan/hyaluronic acid synthesis and known to be involved in the inflammatory response [35], and *Icam2 *encodes a lectin which mediates adhesive interactions during the immune response.

### Nearly half of analyzed glycogenes could be cell homeostasis genes

Among the 276 genes expressed, 181 were invariantly transcribed (Table 1). These constitutively expressed genes could be divided into three sets, according to their functions. The first set corresponded to genes involved in cell homeostasis, the second to genes involved in myogenic cell homeostasis and the third to myogenic genes that could probably undergo a late modification to their expression. In this respect, most of the genes encoding proteins involved in *N*-glycan precursor synthesis and present on our mouse glycogenome TLDA were homeostasis cell genes and were constitutively expressed. *Alg2, Alg3, Alg9, Alg12 *(mannosyltransferase genes) and *Alg6 *(a glucosyltransferase gene), which are responsible for *N*-glycan precursor synthesis, were expressed without any significant variations. This was also the case for *Dpia3 *(or *Erp57*), an ER chaperone-encoding gene involved in disulfide bond formation [36]. The second set of genes, although constitutively expressed during the first 72 h of differentiation, could have crucial functions at all stages of myogenesis. The myogenic factor *MyoD*, or the sialidase gene *Neu3 *are representative of this group [37]. Finally, the expression of the third set of genes may be modified after 72 h of differentiation and be required for later stages of myogenesis. For example, the expression of *Pomt1*, encoding an *O*-mannosyltransferase which is known to glycosylate the muscle membrane protein α-dystroglycan linking cytoskeleton actin to ECM components, could be tardily up-regulated [38].

### Glycogenes with significant mRNA variations are sequentially expressed

**Figure 2:**
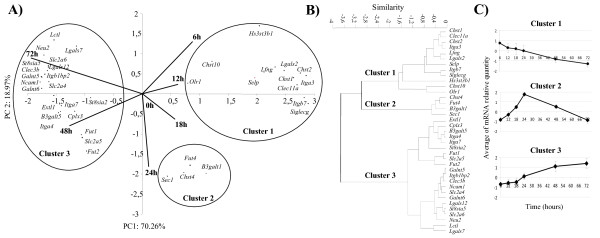
**Expression dynamics of up- and down-regulated glycogenes during the onset of mouse C2C12 differentiation**. (A) According to their expression profiles, the 37 glycogenes expressed with more than-4 fold variations were analyzed using a principal component analysis (PCA). Gene expression profiles in differentiated C2C12 cells were plotted according to the plan defined by the two first principal components, PC1 and PC2. The PC1 axis corresponds to the gradient of gene expression occurring in the time course. Each vector, numbered 0 to 72 h, represents the orientation for genes increasing along each time. In the opposite direction to these vectors are genes whose expression decreased. (B) A dendrogram representation of the hierarchical cluster analysis of the 37 gene expression levels shows three distinct clusters. (C) Expression dynamics of glycogenes were modeled according to their membership cluster.

On the 95 regulated genes, 37 whose expression levels were modified more than 4-fold were retained for further analyses. In order to obtain a global vision of their expression profiles, Principal Component Analysis (PCA) was performed. Its efficiency was excellent since ~89% of information in the data set was recovered on the first ordinate (~70% on component 1 and ~19% on component 2). The localization of each gene in the Figure indicates its expression as a function of differentiation times (6 to 72 h), compared with the precursor state at t = 0 h of differentiation (Figure 2A). The position of a gene in the same direction as a vector indicates an increase of expression. By contrast, the position of a gene in the opposite direction to a vector means that the gene was down-regulated. Because of their reduced sizes, 12 h and 18 h vectors were only weakly informative.

Gene clustering was performed using the Euclidean distances calculated with their coordinates on the first plan of PCA. This clearly highlighted three groups (Figure 2B). The first contained 12 genes, the second four and the third 21. The myogenic marker *Myf5 *was classified in cluster 2, *MyoD *and *myogenin *in cluster 3 (data not shown); *Mrf4 *was not clustered since it was not expressed during the first 72 h of differentiation. mRNA levels in the cluster 1 displayed a general tendency to decrease that was more pronounced towards the end of the time course (Figure 2C). Cluster 2 included genes with a peak mRNA expression at 24 h of differentiation. Genes in cluster 3 had expression profiles opposite to those of cluster 1 because these expressions increased and became more important at the end of the time course (Figure 2C).

**Figure 3:**
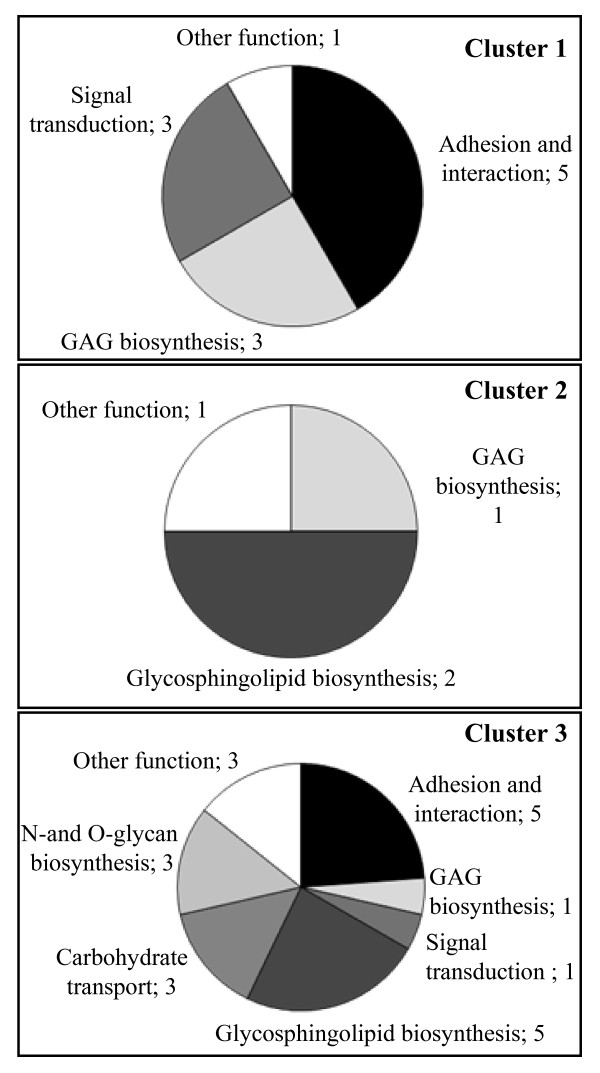
**Cell functions in which regulated glycogenes are involved**. The function assigned to each gene was extracted from the Kegg Pathway database [64]. Numbers indicate how many genes are concerned for each function, one gene being able to be involved in different processes. "Other functions" corresponds to functions unrelated to developmental processes, such as exocytosis and apoptosis.

The 37 highly regulated glycogenes were examined according to the activity/function of the enzymes they encode. Only their functions linked to myogenesis were considered (Figure 3). Functions unknown or unrelated to myogenesis, such as intracellular transport, were grouped in "other function". In the light of the literature, several functions could be assured by one protein. Genes in cluster 1 encoded proteins mainly involved in cell adhesion and interaction, GAG biosynthesis and signal transduction. The down-regulation of most of them could be required for the early mechanisms of myogenesis, especially for the switch from a proliferative to a quiescent state and then to a differentiated state. The four genes in cluster 2 were mainly involved in glycosphingolipid and GAG biosynthesis (Figure 3). These functions suggest early rearrangements of the plasma membrane and ECM, leading to the first fusion events. Among the up-regulated genes in cluster 3, some genes were also involved in glycosphingolipid biosynthesis while the others encoded proteins that were mostly implicated in cell adhesion and interaction and in intracellular biological functions. These functions were consistent with the fusion events leading to myotube formation and maturation beyond 48 h of serum deprivation.

With regards the sequential expression of ≥ 4-fold variant glycogenes and the function of encoded proteins, the early differentiation of C2C12 cells seemed mainly to require: (i) the specific expression of molecules involved in cell signaling and a modification to ECM composition, (ii) the expression of CAMs, and (iii) qualitative and/or quantitative modifications to plasma membrane glycoconjugates.

### Cell signaling and GAGs sulfation contribute to the initiation of myogenesis

The functions assured by some down-regulated genes in cluster 1 suggested an involvement of cell signaling in myogenic differentiation. The commitment of C2C12 cells to the myogenic or adipogenic lineage is controlled by specific transcription factors. Myogenesis is regulated by MRFs [4], while adipogenesis is controlled by PPAR-γ and the C/EBP families of transcription factors [39,40]. The *Olr1 *gene encodes a lectin which is activated by PPAR-γ signaling [41]. The down-regulation of *Olr1 *is consistent with the commitment of C2C12 to myogenic differentiation. Lfng is an enzyme that elongates *O*-fucose on some EGF-like domains of the Notch receptor. It belongs to the Fringe family [42] and acts as a modulator of the Notch signaling pathway [43]. It also influences cell fate during embryonic development [44]. Given the involvement of Notch in the myogenic process [45], *Lfng *down-regulation in differentiating C2C12 cells argues for the involvement of Lfng in myogenic differentiation. Interestingly, among the up-regulated genes in cluster 3, *Lgals12 *encoded the galectin-12 which is required for adipogenic signaling and adipocyte differentiation [46]. This gene is indeed weakly expressed at early stages, but its important transcriptional induction beyond 48 h of differentiation suggests, for the first time, its later implication in myogenesis.

GAGs are known to have many biological functions, including cell adhesion, migration and signaling [47]. Three sulfotransferase genes from cluster 1 (*Chst1, Chst2 *and *Hs3st3b1*) are known for GAG sulfation. Chst1 and Chst2 are involved in the sulfation of keratan GAG and Hs3st3b1 in that of heparan GAG. Because *Hst3st3b1 *is the only gene in cluster 1 which was up-regulated at an early stage (Table 2), heparan GAG could become preferentially sulfated. Moreover, the *Extl1 *gene in cluster 3 encoded a glycosyltransferase that contributes to heparan/heparin sulfate biosynthesis. Thus when C2C12 cells differentiate, they seem to undergo a switch from the sulfation of keratan GAG to the predominant sulfation of heparan GAGs. Such a modification has not previously been reported in myogenesis and it could contribute to the activation of myogenesis. Keratan sulfate GAG may have anti-adhesive properties [48] that are obviously incompatible with up-coming myoblast fusion events during myogenic differentiation.

**Table 2:** Deregulated glycogenes during the onset of C2C12 differentiation.

			**mRNA Relative Quantity according to differentiation time**						
			
**Gene Symbol**	**Protein function**	**Cluster number**	**0 h**	**6 h**	**12 h**	**18 h**	**24 h**	**48 h**	**72 h**
*Lfng*	GTase	1	1	-1.86	-2.19	-2.36	-3.04	**-5.18**	**-5.53**
*Clec11a*	Lectin	1	1	-1.28	-1.67	1.05	-1.81	**-4.86**	**-5.24**
*Itga3*	Lectin	1	1	1.25	-1.15	-1.14	-1.51	-2.55	**-4.17**
*Itgb7*	Lectin	1	1	-1.27	-1.38	-1.52	-1.46	**-10.31**	**-27.41**
*Lgals2*	Lectin	1	1	-1.49	-2.69	-2.84	-3.37	**-6.38**	**-10.55**
*Olr1*	Lectin	1	1	-3.31	**-5.40**	**-8.14**	**-6.10**	**-8.38**	**-8.74**
*Selp*	Lectin	1	1	**-4.82**	**-7.69**	**-10.04**	**-9.78**	**-15.30**	**-38.60**
*Siglecg*	Lectin	1	1	1.22	1.20	1.44	1.20	-3.34	**-6.88**
*Chst1*	Sulfotransferase	1	1	-1.22	1.38	-1.52	-1.95	-2.61	**-5.20**
*Chst2*	Sulfotransferase	1	1	1.02	-1.70	-1.70	-2.20	**-5.54**	**-8.52**
*Chst10*	Sulfotransferase	1	1	-1.69	-3.77	**-4.11**	-2.69	-3.43	-3.77
*Hs3st3b1*	Sulfotransferase	1	1	**5.85**	3.35	2.67	1.27	1.11	1.57

*B3galt1*	GTase	2	1	0.81	1.29	2.01	**4.87**	1.69	0.68
*Fut4*	GTase	2	1	1.25	1.19	1.87	**4.31**	1.87	1.06
*Sec1*	GTase	2	1	-1.69	1.22	2.35	**7.21**	**4.04**	-1.37
*Chst4*	Sulfotransferase	2	1	0.92	1.52	2.95	**6.30**	2.48	1.08

*Lctl*	Glycosidase	3	1	1.15	-1.88	-3.04	-3.10	2.01	**4.12**
*Neu2*	Glycosidase	3	1	1.00	-1.18	1.10	1.00	3.63	**18.97**
*B3galt5*	GTase	3	1	-1.13	1.40	3.19	**5.91**	**6.34**	**16.14**
*Extl1*	GTase	3	1	-1.80	1.49	-2.14	1.73	**46.03**	**9.90**
*Fut1*	GTase	3	1	-1.14	2.16	2.20	**6.11**	**24.61**	**4.70**
*Fut2*	GTase	3	1	-2.00	1.46	1.75	**5.26**	**5.73**	3.02
*Galnt5*	GTase	3	1	-1.76	**-7.21**	**-7.36**	2.01	3.50	**25.31**
*Galnt6*	GTase	3	1	-1.25	1.20	1.08	2.24	**34.32**	**28.52**
*St8sia2*	GTase	3	1	1.90	2.09	2.18	3.05	3.11	**4.08**
*St8sia5*	GTase	3	1	**18.06**	-1.13	1.05	**27.43**	**264.72**	**1345.50**
*Clec3b*	Lectin	3	1	-1.89	**-6.66**	-1.56	-1.22	1.64	**4.84**
*Cplx3*	Lectin	3	1	**9.55**	**12.32**	**2.35**	**16.42**	**219.09**	**36.18**
*Itga4*	Lectin	3	1	1.23	1.74	2.32	**4.57**	**12.41**	**9.28**
*Itga7*	Lectin	3	1	1.37	1.63	1.90	2.67	**4.84**	**4.51**
*Itgb1bp2*	Lectin	3	1	-1.01	1.46	-1.75	2.10	**41.75**	**49.78**
*Lgals12*	Lectin	3	1	**-22.54**	-1.48	**-20.60**	**-13.28**	**11.47**	**40.29**
*Lgals7*	Lectin	3	1	2.44	2.69	1.38	1.81	3.68	**6.63**
*Ncam1*	Lectin	3	1	-1.10	-1.04	1.09	1.41	**7.38**	**8.09**
*Slc2a4*	Sugar carrier	3	1	1.09	2.11	2.24	1.81	**11.97**	**14.68**
*Slc2a5*	Sugar carrier	3	1	1.60	2.62	**5.92**	**14.15**	**10.40**	**11.05**
*Slc2a6*	Sugar carrier	3	1	2.28	1.55	1.23	2.28	**13.71**	**22.33**

Only genes whose expression levels are modified more than 4-fold (bold type) for at least one time course point are presented. Their symbols, functions, PCA clusters and relative quantities of mRNA at each kinetic time point are given.

### CAMs, glycosphingolipids and glycoproteins of the C2C12 plasma membrane appeared to be reshaped for cell fusion

Myoblast fusion into myotubes requires cell interactions. Ten highly regulated glycogenes are involved in cell adhesion (Figure 3). Among the genes in cluster 1, four encoded lectins (*Itga3*, *Itgb7*, *Siglecg *and *Selp*) and one a sulfotransferase (*Chst10*). These five genes have been described in different developmental processes. For example, Itgα3 associated with Itgβ1 have been shown to mediate the migration of endothelial cells and angiogenesis [49]. In the present case, the down-regulation of *Itgα3 *may have been linked to the arrest of myoblast migration and proliferation. In addition, five lectin genes encoding for three integrins (Itgα4, Itgα7 and Itgβ1bp2), one galectin (Lgals7) and Ncam1, belonged to up-regulated genes (Cluster 3). Most of them have important functions in myogenesis: NCAM1 in myoblast fusion [23,24], melusin (encoded by *Itgb1bp2*) in the maturation and/or organization of muscle cells [50], and Itgα7 (with Itgβ1) in myogenesis [51,52]. Up-regulation of these CAM-encoding genes, combined with the down-regulation of the four genes in cluster 1 mentioned above, also suggests a potential switch of CAM during myogenic differentiation.

**Figure 4:**
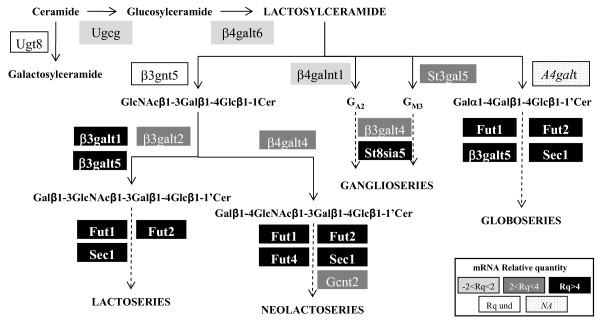
**Schematic representation of glycosphingolipid synthesis pathways**. The enzymes responsible for the main steps of glycosphingolipid biosynthesis are indicated. The expression levels (relative quantity of mRNA) of the corresponding genes are reported. NA: Not Analyzed; Rq: Relative quantity; Und: Undetermined.

Cell fusion obviously requires a modification to the quality and quantity of glycans in plasma membrane glycolipids and glycoproteins. Three genes in cluster 2 (*β3GalT1*, *Fut4 *and *Sec1*) encoded glycosyltransferases implicated in glycosphingolipid biosynthesis (Figure 4). *β*3GalT1 is responsible for synthesizing the precursor of lactoseries glycolipids. Fut4 and Sec1 are involved in the terminal fucosylation of lacto and/or neo-lactoseries glycolipids. Four other genes involved in these different biosyntheses were found in cluster 3 (Figure 3). They encoded two other fucosyltransferases, a sialyltransferase and a galactosyltransferase. The sialyltransferase is involved in ganglioside synthesis, while the three other enzymes are required for lacto and/or neo-lactoseries glycolipid biosynthesis. For glycoproteins, three genes in cluster 3 were revealed: *Galnt5 *and *Galnt6 *encoded *O*-glycan synthesis proteins and *St8sia2 *a sialyltransferase involved in the biosynthesis of Ncam1 polysialic acid (PSA). The latter bears polysialylated *N*-glycans and mucin type *O*-glycans on a muscle-specific domain which is involved in myoblast fusion [24]. The up-regulation of these three genes was in good agreement with the findings of the previous study. Therefore, myoblast fusion may require some glycosphingolipid rearrangements and/or terminal modifications (as fucosylation and sialylation) to glycans of membrane glycoproteins and glycolipids.

### G_M3 _ganglioside levels increase in differentiating C2C12 cells

**Figure 5:**
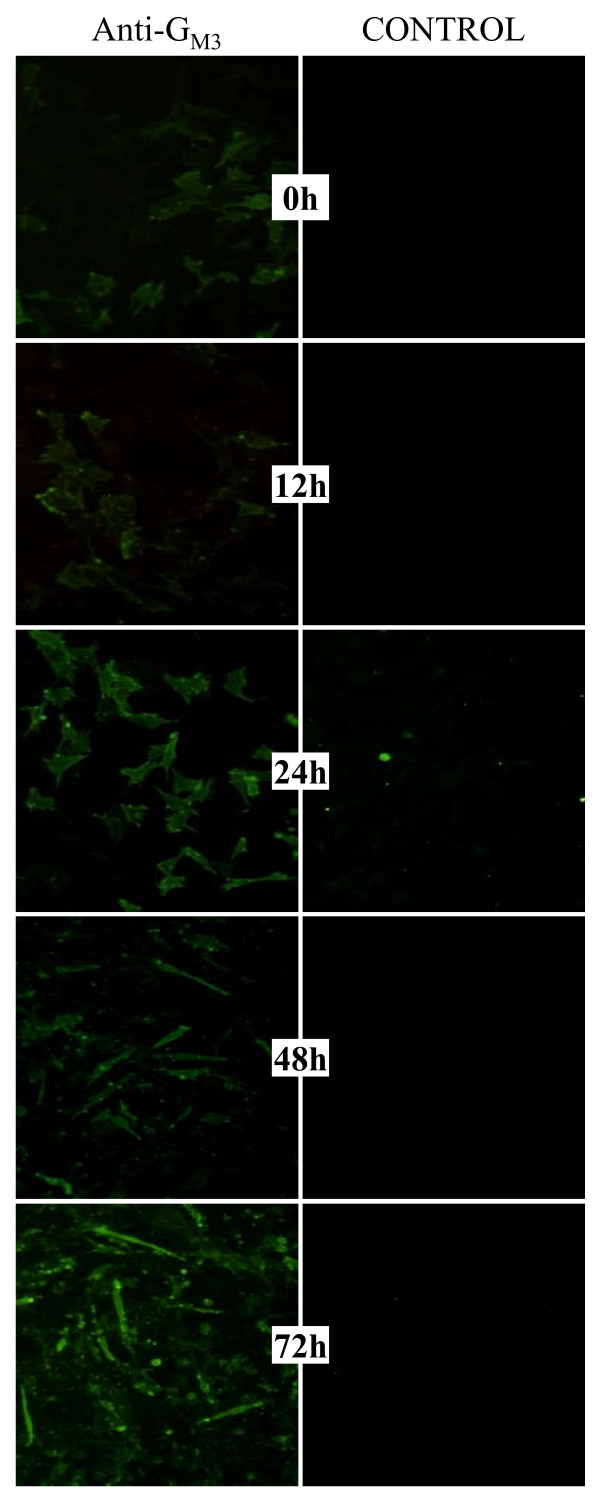
**G_M3 _ganglioside staining in differentiating C2C12 cells**. Cells were labeled with an anti-G_M3 _antibody. This primary antibody was detected by a secondary antibody coupled to FITC. Isotypic controls for each differentiation time point are given.

In order to confirm some of these membrane glycoconjugate rearrangements, glycolipids were considered for further analyses. According to their metabolic pathways and gene expression patterns, lactosylceramid seemed to be preferentially synthesized when compared to galactosylceramid (Figure 4). Indeed, the *Ugt8 *gene was weakly expressed (Ct>33), while the *Ugcg *and *β4galt6 *genes were strongly expressed (Ct<25). Lactosylceramid is the common precursor of four biosynthesis pathways. The expression levels of the analyzed genes implicated in these pathways indicated that some compounds could be preferentially synthesized and/or reshaped. Among these, only G_M3 _(and its derivatives) could be enhanced because the *St3gal5, β3GalT4 and St8sia5 *genes were up-regulated (Figure 4). In order to test this hypothesis, immuno-cyto-staining was used to analyze G_M3 _gangliosides on differentiating myoblasts (Figure 5). Only a few myoblasts are positively stained at 0 h and 12 h of differentiation. Beyond 24 h, the immunostaining increased, and most of the cells were stained at 48 h and 72 h. This result showed that levels of G_M3 _indeed increased in the plasma membrane during the onset of C2C12 differentiation. Interestingly, beyond 48-72 h of differentiation, cells with stronger staining were mostly elongated and underwent differentiation, which argues for a role of G_M3 _in C2C12 differentiation and fusion.

## Conclusion

Little is known about the importance of glycosylation in myogenesis because of the poor representativeness of glycogenes, *i.e*. ~2% of the genome, and because the weak expression of most of them is not revealed by microarrays. In order to determine how glycosylation could be involved in this process, we used a quantitative real-time RT-PCR technology to analyze the expression of 375 glycogenes representing more than 60% of the mouse glycogenome, during the onset of differentiation of the myogenic C2C12 cell line. The glycogenome includes genes encoding for proteins involved in the transport, synthesis and/or recognition of monosaccharide precursors, glycans and glycoconjugates. This study presents for the first time a focused transcriptomic analysis of the glycogenome during myogenic differentiation.

**Figure 6:**
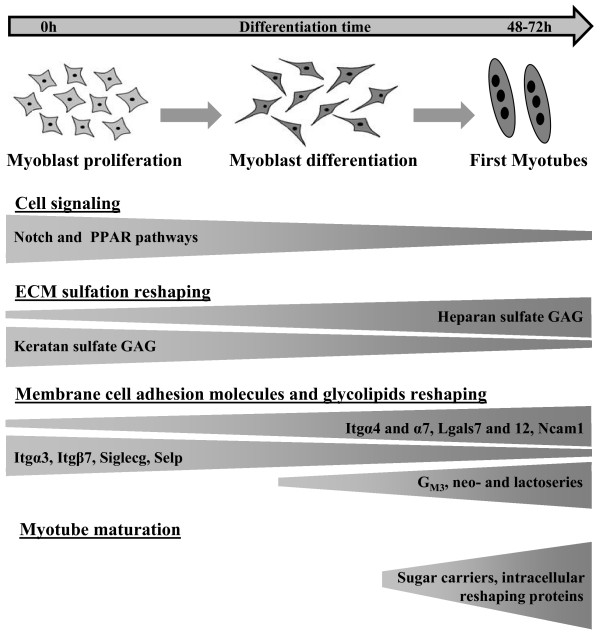
**Hypothetical model of biological processes dependent on highly regulated glycogenes during C2C12 cell differentiation**. On the basis of protein functions encoded by highly regulated glycogenes, four biological and molecular processes are proposed for their implication in C2C12 myoblast differentiation.

Around 75% of the glycogenes thus analyzed was expressed, one third being deregulated by at least 2-fold, showing the importance of glycosylation in this process. Among these deregulated genes, 37 were modified more than 4-fold. Most of these genes (29 genes) had never been associated with myogenesis before. The functions of these 37 glycogenes suggested new roles for glycoconjugates in myogenic differentiation (Figure 6). The initiation of C2C12 differentiation may require specific cell signaling mediated by glycans such as PPAR, and Notch signaling. At the same time, a modification to ECM composition may occur by means of a switch of keratan sulfate GAG to heparan sulfate GAG, in order to promote cell differentiation. Initiation may be followed by the reshaping of membrane glycoconjugates such as cell adhesion molecules, glycolipids and glycoproteins, in order to prepare cells for fusion into myotubes. The lag time in expression between genes encoding CAMs and genes encoding glycolipid synthesis proteins suggests that cell interactions precede membrane glycolipid rearrangements. Finally, initial myotube maturation in late-appearing myofibers involves various intracellular processes dependent on glycosylation. Indeed, a variety of cell functions are associated with proteins encoded by some markedly up-regulated genes. Some of these sugar carriers (GLUT4 and GLUT5) have already been associated with myogenesis [53-55]. Other functions, such as glucose transport by GLUT6 [56], exocytosis by tetranectin (CLEC3b) [57], or the non-lysosomal catabolic pathway by Klotho-related protein (KLrP or LCTL) [58] are suggested by these transcriptional data. Thus, this screening of glycogenome expression provides clues to a clearer understanding of certain stages in myogenesis.

## Methods

### Biological materials

C2C12 mouse myoblasts (strain C3H, American Type Culture Collection (ATCC), Manassas, VA, USA) were cultured in DMEM (Dubelco's modified Eagle's medium, Eurobio, Courtaboeuf, France) supplemented with 10% fetal calf serum (Eurobio), 2 mM L-glutamine, 50 units/mL penicillin and 50 μg/mL streptomycin. Cells were grown to 80% confluence and were differentiated into myotubes with DMEM supplemented with 2% horse serum. After 48 h of differentiation, the medium was changed every day. For each kinetic point analyzed, cells were rinsed briefly with PBS and harvested following trypsinization (1× PBS, 1 mM EDTA, 0.05% (w/v) trypsin).

### RNA extraction and cDNA synthesis

Total RNA from each sample was obtained by anion exchange chromatography (RNeasy mini Kit, Qiagen Inc., Hilden, Germany). The integrity and quantity of total RNA were measured using a micro fluidic-based platform (Agilent 2100 Bioanalyser, Agilent Technologies Inc., Santa Clara, CA, USA). The High Capacity cDNA Archive Kit (Applied Biosystems, Foster City, CA, USA) was used to convert 5 or 10 μg of total RNA into single-stranded cDNA.

### Design of the glycogenome TaqMan Low Density Array (TLDA)

A micro-fluidic card dedicated to quantitative real-time RT-PCR analyses of part of the mouse genome, the 'glycogenome', was designed. The glycogenes thus analyzed encode proteins involved in glycan synthesis or glycan recognition. They were selected from GenBank, CAZY and MGI databases [59-61]. They include glycosidases, glycosyltransferases, sugar carriers and sugar metabolism proteins, translocases, sulfotransferases and lectins. These genes control glycosylation functions which likely regulate myogenesis. When this work started, ~600 corresponding murine genes were listed (Table 1). The TLDA technology used is based on quantitative real-time RT-PCR with TaqMan probes validated by the manufacturer. Among the 600 genes listed, only 389 validated probes were available for gene expression studies . The technology operates on 384 well plates and allows a simultaneous analysis of 375 candidate glycogenes, 9 wells being dedicated to 6 reference genes. Consequently, among the 389 genes, for which validated probes were available, some sulfotransferase genes were not selected in order to preferentially analyze all available genes involved in glycan biosynthesis and not in glycan modification. Thus, for genes encoding sulfotransferases, only 22 probes of the 36 listed were considered (Table 1), reducing to 375 the number of glycogenes analyzed using TLDA, that is ~60% of the mouse genes known to be related to glycosylation.

### Quantitative real-time RT-PCR

The quantity of each mRNA was determined by quantitative real-time RT-PCR on an ABI Prism 7900 Sequence Detector System using TaqMan probe-based chemistry (Applied Biosystems). 6-carboxyfluorescein (FAM) was used as a reporter. The amplification reactions for each gene were performed with 2 ng cDNA for 96-well plates (analysis of myogenic markers) and with 3 ng cDNA for TaqMan Low Density Arrays (TLDA) (analysis of glycogenes). This relative quantification was reliant on the use of several reference genes: *18S RNA, G6pdx, Gapdh, Tcea, Tbp*.

### Data analysis

Gene mRNA expression data were collected and analyzed using SDS 2.2.2 software (Applied Biosystems). The comparative ΔΔCt method was used to quantify the relative abundance of mRNA. This method uses a calibrator sample to enable a comparison of gene expression levels in different samples. During this study, we used time t = 0 h of differentiation as the calibrator sample. The values obtained indicated the changes in expression in the sample of interest by comparison with the calibrator sample after normalization to 18S RNA. Relative quantities were regarded as significant for genes whose Ct (Threshold Cycle) was lower than 33. Genes that were not expressed were given a Ct value of 40 by default.

Relative levels of mRNA in the 37 selected genes were log-transformed and analyzed using Principal Component Analysis (PCA) and hierarchical cluster analysis (HCA) with PAST version 1.78 [62,63] in order to reveal trends in their expression. This mathematical procedure reduces the number of possibly correlated variables (seven dimensions corresponding to the different differentiation times) to a smaller number. Thus, most of the data are projected in a 2D-space defined by the two principal components PC1 and PC2, which are synthetic axes expressing the percentage of data variance. Indeed, PCA extracts the direction where the cloud of values is extended, constituting the first component or principal component (PC1). The next direction (PC2) is orthogonal to the first one. The cloud of points reflects the level of expression of each gene as a function of its position relative to the vectors. Vectors indicate the orientation of variation and correspond to most representative expression profiles. Samples belonging to a same pattern are therefore expected to be grouped in a similar area. The coordinates of each gene on the ordination plan were used to calculate Euclidean distances between all pair-wise combinations. The unweighted pair-group average was taken as an agglomeration method to construct the Hierarchical Component Analysis.

### Immuno-cyto-chemistry

C2C12 cells were grown on glass cover-slips. After removing the medium, the cells were washed twice with PBS and fixed for 15 min in 4% paraformaldehyde. After two washes of 5 min each in 1× PBS, the cells were further incubated for 1 h with a blocking solution (1× PBS with 10% fetal bovine serum (Eurobio)), and labeled with an anti-G_M3 _primary antibody (Seikagaku Corporation, Japan) diluted 1/100 in blocking solution for 1 h at room temperature. A control was performed using cells incubated with a mouse isotypic IgM (Santa Cruz, CA, USA) at the same concentration as the anti-G_M3 _primary antibody. The cells were rinsed in 1× PBS, incubated for 1 h with an FITC-conjugated secondary antibody (Sigma-Aldrich, Saint Quentin Fallavier, France) and then washed 3 times for 5 min with 1× PBS. The cover-slips were washed in PBS and mounted on glass slides. The cells were then observed under an Olympus epifluorescence microscope.

## Abbreviations

CAM: Cell Adhesion Molecule; ECM: ExtraCellular Matrix; GAG: GlycosAminoGlycan; HCA: Hierarchical Cluster Analysis; Itg: Integrin; LIM: protein containing a cystein-rich domain described in Lin-11, Il-1 and Mec-3 proteins; MRF: Myogenic Regulatory Factor; Ncam: Neural cell adhesion molecule; PCA: Principal Component Analysis; PPAR: Peroxysome Proliferator-Activated Receptor.

## Authors' contributions

MJ carried out cell cultures, mRNA extractions, RT-PCR quantification, immuno-cyto chemistry experiments, participated in data exploitation and was involved in drafting and critically revising the manuscript. AA contributed to the acquisition and interpretation of data concerning validation of the model, to drafting corresponding paragraphs in the manuscript and its critical revision. CL participated in critical revision of the manuscript. AG was involved in interpreting the data and in critical revision of the manuscript. AM participated in the design of experiments, the interpretation of data, drafting and critical revision of the manuscript. FD participated in the design of experiments, the design of glycogene arrays, and the performance of statistical data analyses, data interpretation, drafting the manuscript and its critical revision. All authors have read and have given their final approval of the manuscript.

## Supplementary Material

Additional file 1 **List of glycogenes analyzed by quantitative RT-PCR**. This table presents the list of glycogenes analyzed by TLDA (TaqMan Low Density Array) according to their protein function and family. Their symbol, name, NCBI identification and Applied Biosystems identification (Assay ID) are given. Click here for file

Additional file 2 **Results of glycogenes mRNA quantification during C2C12 differentiation**. This table presents the threshold Cycle (Ct) and Relative Quantification (RQ) values of reference gene and glycogenes for each time course point of C2C12 differentiation. The calibrator sample corresponds to time 0 h of differentiation and the normalization gene to 18S. AV: Aberrant Value; ND: Not Determined. Click here for file
